# EVOTECH^® ^endoscope cleaner and reprocessor (ECR) simulated-use and clinical-use evaluation of cleaning efficacy

**DOI:** 10.1186/1471-2334-10-200

**Published:** 2010-07-09

**Authors:** Michelle J Alfa, Pat DeGagne, Nancy Olson, Iram Fatima

**Affiliations:** 1 Diagnostic Services of Manitoba, 409 Tache Avenue, Winnipeg, MB, R2H 2A6, Canada; 2 St. Boniface Research Centre, 351 Tache Avenue, Winnipeg, MB, R2H 2A6, Canada; 3 University of Manitoba, Winnipeg, MB, 745 Bannatyne Avenue, Room 543 Basic Medical Sciences Building, R3E 0J9, Canada

## Abstract

**Background:**

The objective of this study was to perform simulated-use testing as well as a clinical study to assess the efficacy of the EVOTECH^® ^Endoscope Cleaner and Reprocessor (ECR) cleaning for flexible colonoscopes, duodenoscopes, gastroscopes and bronchoscopes. The main aim was to determine if the cleaning achieved using the ECR was at least equivalent to that achieved using optimal manual cleaning.

**Methods:**

Simulated-use testing consisted of inoculating all scope channels and two surface sites with Artificial Test Soil (ATS) containing 10^8 ^cfu/mL of *Enterococcus faecalis, Pseudomonas aeruginosa *and *Candida albicans*. Duodenoscopes, colonoscopes, and bronchoscopes (all Olympus endoscopes) were included in the simulated use testing. Each endoscope type was tested in triplicate and all channels and two surface sites were sampled for each scope. The clinical study evaluated patient-used duodenoscopes, bronchoscopes, colonoscopes, and gastroscopes (scopes used for emergency procedures were excluded) that had only a bedside flush prior to being processed in the ECR (i.e. no manual cleaning). There were 10 to 15 endoscopes evaluated post-cleaning and to ensure the entire ECR cycle was effective, 5 endoscopes were evaluated post-cleaning and post-high level disinfection. All channels and two external surface locations were sampled to evaluate the residual organic and microbial load. Effective cleaning of endoscope surfaces and channels was deemed to have been achieved if there was < 6.4 μg/cm^2 ^of residual protein, < 1.8 μg/cm^2 ^of residual hemoglobin and < 4 Log_10 _viable bacteria/cm^2^. Published data indicate that routine manual cleaning can achieve these endpoints so the ECR cleaning efficacy must meet or exceed these to establish that the ECR cleaning cycle could replace manual cleaning

**Results:**

In the clinical study 75 patient-used scopes were evaluated post cleaning and 98.8% of surfaces and 99.7% of lumens met or surpassed the cleaning endpoints set for protein, hemoglobin and bioburden residuals. In the simulated-use study 100% of the Olympus colonoscopes, duodenoscopes and bronchoscopes evaluated met or surpassed the cleaning endpoints set for protein, and bioburden residuals (hemoglobin was not evaluated).

**Conclusions:**

The ECR cleaning cycle provides an effective automated approach that ensures surfaces and channels of flexible endoscopes are adequately cleaned after having only a bedside flush but no manual cleaning. It is crucial to note that endoscopes used for emergency procedures or where reprocessing is delayed for more than one hour MUST still be manually cleaned prior to placing them in the ECR.

## Background

Cleaning and disinfection of flexible endoscopes presents a significant challenge to reprocessing personnel because of the pressure to quickly turn around the patient-used endoscope so that it is ready for the next patient. In addition to pressure on staff to rush the process there is also the challenge of how well trained and competent the reprocessing personnel are. Current guidelines [1-3] recommend that specific and thorough training be provided initially as well as on an ongoing basis as technology is rapidly changing and adequate training of reprocessing staff on newly acquired models is often overlooked. The manual cleaning phase is a critical part of the reprocessing protocol and is prone to errors [1-11]. These human errors may include; failing to clean channels because staff were not aware of them, failing to properly assess if channels are blocked or leaking, or not flushing adequate fluid volumes through all channels. Puszko [12] demonstrated that manual flushing of channels during the cleaning phase results in potential for repetitive strain injuries in reprocessing personnel and that using flushing pumps provided a more reliable way to ensure adequate fluid volumes were consistently used. The greatest concern when errors are made is that high-level-disinfection (HLD) may be inadequate thereby allowing infectious organisms to survive and be transmitted to the next patient that the endoscope is used on. Infection transmission and chemical colitis associated with improper reprocessing of flexible endoscopes still is a concern [5,13-17]. Despite the advent of automated endoscope reprocessors that have "wash cycles" as part of the whole process, there are a number of studies that question the cleaning efficacy of such cycles [18-21]. Recent simulated-use evaluation of the washing provided by the Reliance Endoscope Processing System indicated that it provided cleaning that was equivalent to manual cleaning [22] but that clinical testing was needed.

The EVOTECH^® ^Endoscope Cleaner and Reprocessor (ECR) has received FDA clearance for cleaning claims that eliminate the need for manual cleaning prior to High Level Disinfection (HLD). Despite this, the Society of Gastrointestinal Nurses and Associates (SGNA) has alerted users in 2007 and again in 2009 that manual cleaning should be continued until clinical testing data is available to confirm that the ECR can provide reliable cleaning without the full manual cleaning process [23,24].

The primary objective of this study was to use simulated-use testing as well as a clinical study to evaluate the efficacy of the ECR in removing bioburden and organic material from the channels and surfaces of flexible endoscopes.

## Methods

### EVOTECH^® ^Endoscope Cleaner and Reprocessor (Hereafter referred to as the ECR)

The ECR (Advanced Sterilization Products, Irvine, CA) was located in the endoscopy clinic of a large tertiary care hospital. The ECR consists of two independently operated endoscope reprocessing basins controlled by a single processor, with sub-systems that monitor and control temperature, pressure, fluid flow rate and the water, detergent, disinfectant and alcohol delivery systems. The ECR was fed with softened tap water as per the manufacturer's instructions. As per the manufacturer's instructions the valves were manually cleared prior to placing in the ECR. A unique software program was made available in the ECR that allowed the cycle to be stopped at the end of the cleaning phase such that samples could be collected from the processed endoscopes prior to HLD (Note; this cycle is not commercially available) CIDEZYME^®^-GI Detergent (Advanced Sterilization Products Irvine, CA) was the enzymatic detergent used in the ECR cleaning cycle at 1:185 dilution (as per manufacturer's recommended use-dilution). As indicated in the ECR manufacturer's cycle parameters, once the reprocessing cycle has been initiated, the first step is the leak test. If the pressure does not reach the target and stabilize or if a substantial leak is detected, the cycle cancels. The next step is a pre-rinse where the basin is filled with ambient temperature filtered water (all filtered water in the cycle is achieved using 0.2 μm filtration) to remove any gross contamination in the channels. The water is pumped through the endoscope channels and drained. The pre-rinse consists of cool water followed by a warm water rinse. This is followed by the wash stage where the basin is filled with filtered water to which CIDEZYME^®^-GI detergent is added. The water temperature during the entire cleaning phase is held at 35°C (if the water temperature falls below this the cycle will be aborted and an error message displayed). The detergent solution is circulated for 3 minutes and then drained and followed by a rinse. Filtered air is blown through the channels. An automated blockage test in then performed on each individual channel with specific criteria for each channel to determine pass or fail. After the blockage test the rinse stage begins during which the basin is filled with filtered water at 45°C and circulated through the channels for at least 30 seconds then drained. The ECR then disinfects the endoscope by perfusing all lumens with a 0.055% in-use concentration of CIDEX^® ^OPA concentrate solution that is held at 50-55°C and circulated through the channels for 5 minutes. At the end of the HLD cycle the disinfectant solution is drained and filtered air is blown through the channels. After HLD, two rinses are performed using filtered water. After the final rinse, filtered air is blown through the channels. Following the cleaning and HLD cycle, there is an optional flush with 70% isopropyl alcohol. Finally the leak test is repeated.

The entire cleaning phase (includes rinses and detergent exposure phase) is approximately 10 to12 minutes long. CIDEZYME^®^-GI detergent is the only one recommended by the ECR manufacturer and the results obtained in this study apply to the ECR only when this detergent is used.

### Cleaning Study overview

The study was carried out in two phases with Phase I being a clinical in-use study and Phase II being a simulated-use study. These two phases were necessary to ensure that the ECR cleaning cycle was thoroughly evaluated under actual patient-use conditions (where soiling levels were reflective of what happens in actual usage but where the level of soiling cannot be controlled, i.e. worst-case clinical conditions) as well as under controlled inoculation studies where all channels and surface test sites could be reproducibly inoculated with high bioburden and organic levels (i.e. worst-case simulated-use conditions that are representative of worst-case clinical conditions).

The accepted benchmark(s) for adequate cleaning are still under debate and the only currently published benchmarks for residual organic and bioburden levels in flexible endoscopes that are based on clinical measurements are those referred to in the AAMI ST35 [8] and TIR30 [25] which are based on the work published by Alfa et al [26,27]. These published benchmarks were used in this current study to assess the adequacy of cleaning for both Phase I and II studies and were set at; < 6.4 ug/cm^2 ^of residual protein, < 1.8 ug/cm^2 ^of hemoglobin and < 4 log_10 _viable bacteria/cm^2 ^[25,28]. As such those samples collected from the endoscope channel or surface after ECR cleaning in the current study that had organic and bioburden levels lower than the stated benchmarks were considered to have been adequately cleaned (i.e. equivalent to routine manual cleaning). Previously reported testing has shown that manual cleaning of endoscopes tested after routine cleaning of patient-used endoscopes [27] as well as those tested after inoculation using ATS in simulated-use studies do achieve these benchmarks [26]. The step by step manual cleaning protocol that was used for comparison to the ECR cleaning cycle took experienced research personnel between 14 minutes (bronchoscopes) to 25 minutes (side-viewing duodenoscope) to complete. The 14 step manual cleaning process (no flushing pumps used in this protocol) is clearly described in Alfa et al [26].

### Microbial cultures

The bacterial strains used for Phase II simulated-use testing included; *Enterococcus faecalis *(EF) ATCC 29212, *Pseudomonas aeruginosa *(PA) ATCC 27853, and *Candida albicans *(CA) ATCC 14053. All microorganisms were stored as frozen skim milk stocks at -70°C and were passaged three times before use in any testing. All microorganisms were grown on Tryptone Soya Agar + 5% Sheep Blood (BA) (Oxoid Inc. cat #MP2012).

### Organic Test Soil

The organic challenge was provided using Artificial Test soil-thickened (ATS-T; US Patent # 6,447,990; inventor Dr. Michelle Alfa) which was prepared fresh, stored at 4°C and used within one month of preparation as described by Alfa et al [26,22]. This test soil was formulated to reflect the worst-case levels of organic material based on data obtained from patient-used flexible endoscopes [26], Patent # 6,447,990] prior to bedside pre-cleaning or manual cleaning. The three test organisms were added to achieve a final concentration of approximately 10^8 ^cfu/mL in ATS-T. The main components in ATS-T include; whole sheep blood, serum, physiological solution and a thickening agent. The protein, carbohydrate, haemoglobin and bioburden levels in ATS-T were representative of the worst-case levels found in patient-used flexible endoscopes [22].

### Effect of CIDEZYME^®^-GI detergent on viability of test microorganisms

CIDEZYME^®^-GI was prepared at its use-dilution (1:185 dilution) using sterile tap water as well as sterile tap water controls were held in a heating block at 35°C. Test organisms were added to achieve a final concentration of approximately 10^6 ^cfu/mL of each organism. Samples were collected after 1, 3, 5 and 10 minutes exposure and viable counts performed using the spread-plate technique. Testing over the 10 minutes timeframe was selected because the length of exposure to enzymatic detergent in the ECR is approximately 3 minutes.

### Phase I Clinical Use study

The clinical use (CU) study consisted of patient-used flexible endoscopes used for a variety of non-emergency procedures. Normally flexible endoscopes used on patients receive "bedside pre-cleaning" and then are transported to the reprocessing area where they are leak tested and then have "manual cleaning" followed by HLD. Bedside pre-cleaning consisted of flushing enzymatic detergent solution (Renuzyme used at 1:32 use-dilution) through all channels and wiping off the exterior of the insertion tube using a cloth moistened with the same enzymatic detergent. The manual cleaning consisted of immersing the scope in use-dilution enzymatic detergent (Renuzyme used at 1:64 use-dilution), brushing of the suction-biopsy channel (and any other channel that the endoscope manufacturer recommended brushing), flushing all channels with enzymatic detergent while immersed followed by transfer and immersion in a basin of tap water and flushing all channels while immersed.

The ECR manufacturer states that flexible endoscopes used for emergency procedures or that have transit times beyond one hour require full manual cleaning prior to placement in the ECR. For this study flexible endoscopes used for emergency procedures were excluded and all patient-used endoscopes evaluated had transit times of less than one hour. The patient-used endoscopes evaluated in this study were given the usual bedside pre-cleaning with enzymatic detergent solution and then placed directly into the ECR. The CU study involved 15 bronchoscopes where 10 received cleaning only, 5 received cleaning and high level disinfection (HLD). In addition there were 20 colonoscopes, 20 duodenoscopes and 20 gastroscopes with 15 of each scope type receiving cleaning only and an additional 5 that received cleaning and HLD (Table 1). Both FDA guidance documents [28] and AAMI [25] recommend that testing be performed to assess each phase of the washer-disinfector cycle as well as the entire process. Therefore, this approach was used for the current study and samples were collected to evaluate the cleaning phase as well as the entire cleaning and HLD cycle of the ECR.

**Table 1:** Summary of scopes and samples tested for Clinical-use and Simulated-use evaluations

Site	Colonoscope	Gastroscope	Bronchoscope	Duodenoscope
	(N = 20)**	(N = 20)**	(N = 15)**	(N = 20)**
**Clinical-use Testing:**				

Surface				
S1 (insertion tube*)	√	√	√	√
S2 (control head*)	√	√	√	√

L1: Suction biopsy channel (umbilical to distal end)	√	√	√	√
	**[364.5 cm**^**2**^**]**^**1**^	**[255.7 cm**^**2**^**]**	**[53.2 (or 58.7) cm**^**2**^**]**	**[402 cm**^**2**^**]**

L2: Air/water channel (umbilical to distal end)	√	√		√
	**[377 cm**^**2**^**]**	**[ 269 (or 254) cm**^**2**^**]**		**[363 cm**^**2**^**]**

L3: Auxiliary water channel	√			
	**[175.3 cm**^**2**^**]**			

L4 Elevator wire channel				√
				**[43.4 cm**^**2**^**]**

Samples collected per endoscope	5	4	3	5

Simulated-use Testing; each scope type tested in triplicate***				

**Site**	**Colonoscope**	**Gastroscope**	**Bronchoscope**	**Duodenoscope**
	**(N = 3)**	**Not done**	**(N = 3)**	**(N = 3)**

Surface				
S1 (insertion tube)****	√		√	√
S2 (control head)****	√		√	√

L1: Suction biopsy channel (From umbilical to distal end)	√		√	√
	**[358.7 cm**^**2**^**]**		**[53.2 cm**^**2**^**]**	**[307.5 cm**^**2**^**]**

L2: Air/water channel (From umbilical to distal end)	√			√
	**[345 cm**^**2**^**]**			**[213.9 cm**^**2**^**]**

L4 Elevator wire channel				√
				**[34 cm**^**2**^**]**

Samples per scope	4		3	5

* Surface area sampled was 1 cm^2 ^for each site

** Clinical use testing: Colonoscope, Gastroscope and Duodenoscope: 15 tested after cleaning only, 5 tested after cleaning and HLD, Bronchoscope: 10 tested after cleaning only, 5 tested after cleaning and HLD

*** Simulated use testing: Colonoscope, Bronchoscope and Duodenoscope: positive and negative controls as well as ECR cleaning was tested for each scope type in triplicate.

**** Surface area inoculated and sampled was 1 cm^2 ^for each site

^1 ^The calculated surface area was determined using the manufacturer's published lumen diameter and the length of the channel as determined by threading a fishing wire down the channel lumen and measuring the length. Where two values are stated these refer to the two different scope models used for this study.

The flexible video endoscopes evaluated in this study were all Olympus scopes including; bronchscope models BF-P60 and BF-P40, duodenoscope model TJF-160VF, colonoscope models CF-Q180AL and CF-H180AL and gastroscope models GIF-Q180 and GIF-H180 (more details of the dimensions of these specific models of endoscopes can be found on the manufacturer's website). Since the internal surface area of flexible endoscopes is not provided by the endoscope manufacturers, the channel length was measured by threading fishing wire through the channel and this measurement along with the channel inner diameter (which is provided by the scope manufacturers) was used to calculate the surface area for each channel.

The patient-used endoscopes that were processed through the ECR were then fully reprocessed using the routine process of manual cleaning followed by HLD. This additional reprocessing was required by the site as this was considered to be a research study. Research and Ethics approval was obtained and since each scope was double-processed (i.e. research sample collected and then reprocessed by usual endoscopy clinic protocol) informed consent from the patients was not required.

### Phase II Simulated-use Study

The simulated-use (SU) study was performed using all Olympus scopes including; bronchoscope BF P40, duodenoscope JF 1T30 and colonoscope CF 40L. Table 1 outlines the channels and surface sites tested (more details of the dimensions of these specific models of endoscopes can be found on the manufacturer's website). The inner surface area of the channels was calculated as described for the clinical endoscopes. These scopes were only used for research purposes and were not used on any patients. Every channel in the scope being studied was perfused completely with ATS containing 10^8 ^cfu/mL of each test organism as described in Alfa et al [26] Once the channel was perfused, excess inoculum was flushed out using air. Two surface sites (1 cm^2 ^surface area) were then inoculated with 50 μL of ATS-microbe mixture that was spread over a 1cm^2 ^surface area. A template was used to indicate the surface area to inoculate. The inoculated endoscope was allowed to dry for one hour. Negative controls (uninoculated endoscopes) and positive controls (inoculated endoscopes that did not receive any cleaning) were included in the study. This inoculation process has been utilized in the authors research lab since 1999 [26].

### Endoscope sample collection

All channels from SU and CU endoscopes were evaluated by collecting samples using the "flush only" method or the "flush-brush-flush" method described by Alfa et al [22]. The total sample volume was 40 mLs for L1, 20 mLs for L2, 10 mLs for L3 and 5 mLs for L4. The surface sites were sampled as described by Alfa et al [22] by using a pre-moistened swab that was rubbed vigorously over the inoculated 1 cm^2 ^surface area (a template was used to indicate the surface area to sample) and then placed into 2 mLs of sterile reverse osmosis (RO) water. The samples from lumens and surfaces were mixed for 1 min on a vortex mixer, sonicated (Bransonic 1200 ultrasonic bath, Bransonic Canada, Pickering, ON) for three 5 second pulses, and then mixed a second time for 1 min on a vortex mixer. The sampling method was validated using repeated rounds of sample collection to demonstrate that ≥ 95% of the organic and bioburden residuals were recovered in the first round of sample collection and that there was no added value in more extensive sampling. These data are not shown as the testing was done prior to this study and this sampling method has been in use since then.

### Quantitative assays for endoscope samples

The endoscope samples collected were each assayed to determine the protein, and hemoglobin concentrations as well as the bioburden level. The QunatiPro BCA Assay kit which includes a bovine serum albumin protein standard and uses bicinchoninic acid (Sigma, St. Louis, Missouri) was used for protein quantitation. The TMB (3,3',5,5' Tetramethylbenzadine) Liquid substrate system for ELISA (Sigma) was used in conjunction with a 80 mg/dL cyanonethemoglobin standard (Stanbio Laboratory, Boerne, texas) for haemoglobin quantitation. Both the protein and haemoglobin assay were used as per the manufacturer's instructions. The limit of detection (LD) for the protein assay was 0.5 μg/mL and for the haemoglobin assay the LD was 5 μg/mL. These samples were also assayed to determine the level of bioburden using standard spread plate technique. Briefly, viable counts using serial 1:10 dilutions with triplicate spread plates inoculated with 0.1 mLs were performed using BBL™ CHROMagar™ Orientation media as this facilitated differentiating the three different types of microorganisms as they produced different colored colonies in this media. The limit of detection for the viable count assay was 10 cfu/mL. For more detailed description of the methods used please see Alfa et al [26].

### Reprocessing of simulated-use endoscopes

After sample collection was completed all endoscopes were leak tested (Olympus Model MU-1 leak tester) and if no leaks detected they were then fully manually cleaned (following the manufacturer's instructions). A suction Pump (Olympus model SSU-2) and flushing pump (PCI Medical Model EFP-250) were used for this manual cleaning process as per the manufacturer's instructions. Once fully cleaned the research endoscopes were processed through the ASP Automated Endoscope Reprocessor (AER) for HLD using CIDEX^®^OPA Solution.

### Data analysis

As has been done in previously published analysis of AER cleaning [22] efficacy negative controls (baseline) consisted of samples taken from the flexible endoscopes that had been cleaned and high level disinfected (i.e. patient ready endoscopes in the Clinical study and non-soiled endoscopes in the Simulated-use study). All data presented in the tables and figures has been normalized against the corresponding baseline samples (i.e. the average value obtained for clean, disinfected endoscopes was subtracted from the each test value). This is represented by the following equation:

Where:

[***XP***_***N***_] represents the "normalized" amount of residual protein, haemoglobin or bioburden/cm^2 ^for the patient-used scope tested.

XP represents the amount of residual protein, haemoglobin or bioburdern/cm^2 ^after the patient-used endoscope had been cleaned or cleaned and HLD.

BP represents the average amount of residual protein, haemoglobin or bioburden/cm^2 ^for five fully reprocessed endoscopes that were patient-ready but had not yet been used.

The same calculations described for normalization were done for the samples collected during the Phase II Simulated-use study.

This approach was used to account for any low levels of organic residuals that may be present due to routine handling as flexible endoscopes are not stored under sterile conditions. In addition this approach helps confirm that there was no "accumulation" of material over the duration of the study.

## Results

**Figure 1:**
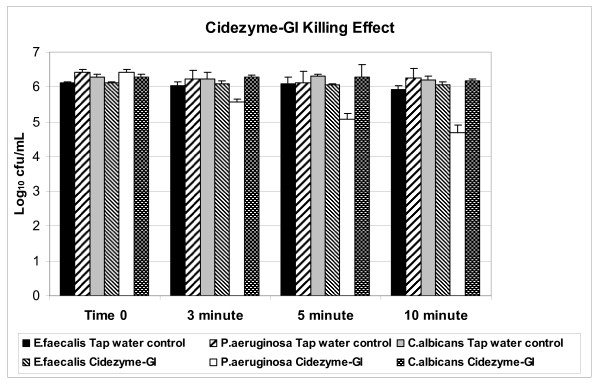
**Effect of CIDEZYME^®^-GI detergent on viability of *E.faecalis, P.aeruginosa *and *C. albicans***. The organisms were suspended in the use-dilution of the detergent of in tap water controls and at the timed intervals noted samples were taken to determine the level of viable bacteria.

Since viable counts were one of the benchmarks being assessed, it is necessary to ensure that the exposure to detergent alone did not contribute significantly to the killing of the test microorganisms. Figure 1 shows the results of this testing. *P. aeruginosa *was the only microorganism affected by exposure to the enzymatic detergent and the net effect was a 1.0 Log_10 _reduction in viability after 3 minutes exposure (length of detergent exposure in ECR). The viability of the other two test organisms was not affected even after 10 minutes of detergent exposure at 35°C.

The clinical-use evaluation was carried out over an eight month period. Bronchoscopes, colonoscopes, duodenoscopes and gastroscopes were evaluated after ECR cleaning only as well as after a full cycle that included HLD using CIDEX^® ^OPA Concentrate in-use (OPAC) Solution. The range of negative control values confirmed that there were no abnormally high levels or protein, haemoglobin or bioburden in any of the patient-ready endoscopes tested. The highest average level detected for negative controls was 9.248 μg/cm^2 ^protein for the S1 (surface) of bronchoscopes, and 0.346 Log_10_cfu/cm^2 ^for bioburden in the L4 channels of duodenoscopes. The haemoglobin level in all negative scopes for all sites was < LD. The protein detected most likely would reflect handling and storage under non-sterile conditions as these protein levels were only detected on surfaces (lumen levels for negative controls for all scope types were all < 6.4 μg/cm^2 ^protein). There were only two surface sites (both S2) from ECR cleaned patient-used endoscopes that were > 6.4 μg/cm^2 ^protein before subtracting the average negative control (these were 8.990 and 6.962 μg/cm^2 ^protein). The ECR cleaning actually removed some of the pre-existing residual surface protein remaining from routine manual cleaning for bronchoscopes at this facility as 5 of 10 negative controls (i.e. 50% of patient-ready bronchoscopes not cleaned by ECR (i.e. received regular manual cleaning) had > 6.4 μg/cm^2 ^protein residuals). The summary of results after the cleaning phase are shown in Table 2 (all data has been normalized against the negative control values as outlined in the Materials and Methods section). There were five replicates of each scope type that were fully processed (ECR cleaning and HLD) after patient use (results not shown in Table 2). Of the 20 endoscopes given full reprocessing there were only two that showed any residuals above the LD. The L1 channel only from one of the five colonoscopes and the L1 channel only from one of the five bronchoscopes had residual bioburden of 0.35 Log_10_cfu/cm^2 ^and 0.30 Log_10_cfu/cm^2^, respectively. All other surfaces and lumens from all fully reprocessed scopes evaluated showed less than the limit of detection for hemoglobin, protein and bioburden.

**Table 2:** Summary Clinical Use Evaluation

Scope Site:	Average of residuals remaining post-cleaning*Average (Standard Deviation) [Range]		
	
	**Protein μg/cm**^**2**^	**Hemoglobin μg/cm**^**2**^	**Bioburden Log**_**1**0 _**cfu/cm**^**2**^
**Bronchoscopes: [N = 10]**			

**S1**	< LD	< LD	< LD

**S2**	0.32 (0.83)	< LD	0.12 (0.26)
	[0 - 2.62]		[0 - 0.67]

**L1**	< LD	< LD	0.03 (0.09)
			[0-0.29]

**Colonoscopes: [N = 15]**			

**S1**	0.26 (0.79)	< LD	0.12 (0.20)
	[0-2.94]		[0 - 0.67]

**S2**	1.04 (2.47)	< LD	0.02 (0.08)
	[0-9.41]		[0-0.33]

**L1**	0.12 (0.12)	< LD	0.04 (0.07)
	[0-0.32]		[0-0.25]

**L2**	< LD	< LD	0.0007 (0.022)
			[0-0.008]

**L3**	< LD	< LD	0.007 (0.023)
			[0-0.09]

**Duodenoscopes: [N = 15]**			

**S1**	0.03 (0.11)	< LD	0.37 (0.94)
	[0-0.44]		[0-3.34]

**S2**	1.28 (3.80)	< LD	0.14 (0.53)
	[0-14.		[0-2.04]

**L1**	0.21 (0.67)	< LD	0.26 (0.97)
	[0-2.60]		[0-3.78]

**L2**	0.043 (0.09)	< LD	0.11 (0.41)
	[0-0.33]		[0-1.58]

**L4**	0.06 (0.11)	< LD	0.81 (1.35)
	[0-0.31]		[0-4.47]

**Gastroscopes: [N = 15]**			

**S1**	1.14 (1.77)	< LD	0.10 (0.30)
	[0-6.44]		[0-1.13]

**S2**	0.63(1.80)	< LD	0.03 (0.06)
	[0-6.99]		[0-0.15]

**L1**	0.07 (0.20)	< LD	0.04 (0.06)
	[0-0.75]		[0-0.24]

**L2**	0.03 (0.10)	< LD	0.11 (0.35)
	[0-0.38]		[0-1.22]

* All values have been "normalized" against the baseline (negative) controls (i.e. the average for the negative controls was subtracted for all test values). The term "LD" indicates the sample concentration was below the limit of detection. The LD for the protein assay was 0.5 μg/mL, for the haemoglobin assay the LD was 5 μg/mL, and for bioburden assay the LD was 10 cfu/mL.

To demonstrate how many were totally compliant with the cleaning benchmarks, Table 3 provides a summary of all the patient-used scopes and all the sites tested that were cleaned only by the ECR. The overall compliance of the ECR cleaning with all benchmarks for surfaces and lumens was > 99%.

**Table 3:** Summary of Patient-used endoscopes; compliance with cleaning benchmarks after ECR cleaning cycle

	Number of sites compliant with cleaning benchmark (All scope types combined)*		
	**Hemoglobin**	**Protein**	**Bioburden**
	**(< 1.8 μg/cm**^**2**^**)**	**(< 6.4 μg/cm**^**2**^**)**	**(< 4 Log_10_/cm**^**2**^**)**

**S1**	53/53 (100%)	52/53 (98%)	55/55 (100%)

**S2**	55/55 (100%)	52/55 (95%)	55/55 (100%)

**All Surfaces, All benchmarks: 322/326 (98.8%)**			

**L1**	54/54 (100%)	54/54 (100%)	54/54 (100%)

**L2**	45/45 (100%)	45/45 (100%)	45/45 (100%)

**L3**	15/15 (100%)	15/15 (100%)	15/15 (100%)

**L4**	15/15 (100%)	15/15 (100%)	14/15 (93%)

**All Lumens, All benchmarks: 386/387 (99.7%)**			

* Note: there were two S1 samples that were not collected from one colonoscope and from one duodenoscope and one L1 sample that were accidentally lost during processing and was not available for analysis so the demoninator value is 53 or 54 instead of 55.

The range of negative control values confirmed that there were no abnormally high levels or protein, haemoglobin or bioburden in any of the simulated-use endoscopes tested. The highest average level detected for negative controls was 4.767 μg/cm^2 ^protein for the S1 (surface) of duodenoscopes, and 0.342 Log_10_cfu/cm^2 ^for bioburden in the S1 sites of duodenoscopes. These data confirmed that despite the extensive soiling used for all channels in this Phase II study that there was no detectable "accumulation" of organic material or bioburden within the channels over the course of the study. Table 4 provides a summary of the simulated-use evaluation using bronchoscopes, colonoscopes and duodenoscopes (all values have been normalized against the negative controls as described in the Materials and Methods Section). Simulated-use testing provides reproducible inoculation of all channels as well as the surface sites to be tested. Unlike clinical-use testing it is possible to have positive controls and to determine the percentage reduction for protein residuals as well as the Log_10 _Reduction Factor (RF) for bioburden. The overall protein reduction achieved by the ECR cleaning was > 99% and the overall bioburden RF was > 4.

**Table 4:** Summary Simulated-use evaluation

	Average of residuals:*					
**Scope Site:**	**Protein μg/cm**^**2**^			**Bioburden Log**_**10**_**/cm**^2**^		
	**Average (STD)**			**Average (STD)**		
	
	**Pos control**	**Post-clean**	**% Reduction**	**Pos control**	**Post-clean**	RF***

**Bronchoscopes: [N = 3]**						

**S1**	1229.6 (133.9)	4.8 (1.5)	99.6%	7.08 (0.23)	< LD	≥ 7.08

**S2**	887.6 (388.6)	2.5 (2.2)	99.7%	7.01 (0.21)	< LD	≥ 7.01

**L1**	517.6 (221.3)	1.4 (1.7)	99.7%	6.71 (0.45)	1.19 (1.10)	5.52

**Colonoscopes: [N = 3]**						

**S1**	1663.6 (246.2)	1.13 (2.4)	99.9%	6.35 (0.37)	< LD	≥ 6.35

**S2**	1677.9 (178.3)	2.01 (0.92)	99.9%	6.99 (0.25)	0.31 (0.25)	6.69

**L1**	437.4 (118.4)	0.23 (0.14)	99.9%	6.54 (0.21)	0.61 (0.69)	5.93

**L2**	458.2 (111.1)	0.17 (0.11)	99.9%	6.46 (0.31)	1.56 (1.14)	4.90

**Duodenoscopes: [N = 3]**						

**S1**	1703.3 (231.8)	< LD	≥ 99.99%	5.95 (0.45)	0.05 (0.08)	5.90

**S2**	1287.9 (115.5)	< LD	≥ 99.99%	6.52 (0.19)	0.01 (0.02)	6.51

**L1**	378.0 (89.6)	0.02 (0.10)	> 99.99%	6.44 (0.19)	0.09 (0.16)	6.35

**L2**	368.9 (71.5)	0.02 (0.04)	> 99.99%	6.59 (0.07)	1.05 (1.24)	5.54

**L4**	136.1 (17.6)	< LD	≥ 99.99%	5.88 (0.65)	1.87 (0.65)	4.01


* Note: The residuals for protein and bioburden for all sites tested met the benchmark for cleaning of < 6.4 ug/cm^2 ^protein and < 4 Log_10 _cfu/cm^2 ^bioburden. Each value represents the average of triplicate tests. All values have been "normalized" against the baseline (negative) controls (i.e. the average for the negative controls was subtracted for all test values). The term "LD" indicates that the sample concentration was below the limit of detection. The LD for the protein assay was 0.5 μg/mL and for the bioburden assay the LD was 10 cfu/mL.

**Average of all three microorganisms together

*** RF; Log_10 _Reduction factor

**Figure 2:**
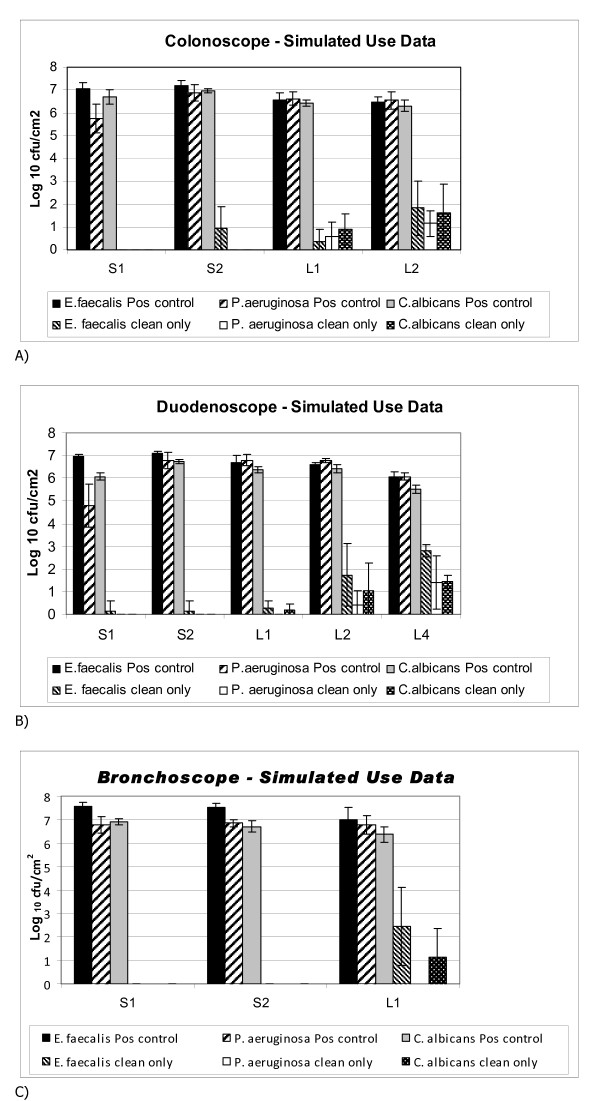
**A summary of the simulated-use testing to assess the reduction in *E. faecalis, P. aeruginosa *and *C. albicans *after ECR cleaning**. All results represent the average and standard deviation for three replicate experiments. S1 and S2 represent samples from 1 cm^2 ^surface sites on the insertion tube and control head, respectively. L1, L2, L4 represent samples taken from the suction-biopsy, air-water and elevator guidewire channels, respectively. [Note; there were no Auxiliary water channels (L3) on the endoscopes used for simulated-use testing.]

Figure 2 provides a summary of the removal of bioburden stratified by organism tested. Although the impact of the detergent may partly explain why *P. aeruginosa *was the most susceptible to removal, the data for the other two test organisms demonstrated that there was significant removal of microorganisms from both lumens and surfaces as neither *E. faecalis *or *C. albicans *were affected by the detergent yet they were effectively removed by the ECR cleaning (4 to 7 Log_10 _reduction in bioburden).

## Discussion

When the Endoscope Cleaner and Reprocessor (ECR) was cleared by the United States Food and Drug Adminstration (FDA) and first marketed, it was the first commercially available endoscope cleaner and reprocessor that had label claims regarding the efficacy of the automated cleaning cycle that allowed elimination of manual cleaning. Despite the desire for automated cleaning of patient-used flexible endoscopes, there was concern that there were no published clinical-use studies for the ECR. The American Society for Gastrointestinal Endoscopy (ASGE) and Society of Gastroenterology Nurses and Associates (SGNA) [24] sent out an alert in 2007 to users regarding the ECR that stated: ***"Members are cautioned about dispensing with manual washing and brushing steps before the capabilities of the new machine are confirmed in independent studies and in clinical practice. Lastly, all currently used machines in the United States are labelled specifically for use only after manual washing with mechanical brushing. Diligence in application of all steps of washing and disinfection remains paramount in the safe delivery of endoscopic services."***

There was major concern regarding whether the bedside flush combined with the cleaning in the ECR was sufficient to provide adequate cleaning. The SGNA alert recommended that sites continue performing manual cleaning until clinical-use data was published. Indeed this concern was reiterated in 2009 by SGNA [23] where they stated: ***"It is necessary to follow all steps for the manual cleaning of the endoscope prior to using an automated reprocessor. No independent confirmatory data are currently available to show that automated reprocessors are able to provide cleaning of endoscopes that is comparable to that of manual washing and brushing."***

The results of our study are the first published data to address this issue. Our clinical use study and simulated-use study clearly demonstrated that the cleaning process used by the ECR provided excellent removal of both organic material (protein and hemoglobin) and bioburden from all flexible endoscopes evaluated. The clinical study by Alfa et al [27] indicated that after complete manual cleaning (before HLD) the range of residual material in the suction/biopsy channels of Olympus bronchoscopes, duodenoscopes and colonoscopes was 0 μg/cm^2 ^to 4.40 μg/cm^2 ^for hemoglobin, 3.51 to 8.55 μg/cm^2 ^for protein and 2.17 to 4.05 Log_10_/cm^2 ^for bioburden. Although the endoscope reprocessing staff in the clinic at the time the 1999 study [27] was conducted were following the Olympus flexible endoscope manufacturer's cleaning instructions they were likely not adhering to it completely as the average time required in the endoscopy clinic for cleaning flexible endoscopes ranged from 5 minutes to 6.5 minutes [27] whereas research personnel who timed all stages took between 14 minutes to 25 minutes to clean the same scope types. At the time of the 1999 [27] study, the endoscopy clinic cleaning protocol was totally manual with no flushing pumps being used.

For simulated-use testing the residuals reported by Alfa et al [22] for optimal manual cleaning by research personnel were lower (maximum residual protein was 4.46 μg/cm^2 ^) compared to the residuals (up to 8.55 μg/cm^2 ^) left after routine manual cleaning of patient-used flexible endoscopes in a busy endoscopy clinic [27]. The data from the current study demonstrated that for patient-used flexible endoscopes the residuals for protein, hemoglobin and bioburden in the suction channel (L1) after the ECR cleaning are substantially better (99.7% met all benchmarks) compared to optimal manual cleaning. Indeed it is clear from the data on manual cleaning of patient-used flexible endoscopes presented by Alfa et al [27] that after routine manual cleaning in an endoscopy clinic there are scopes that may have > 6.4 μg/cm^2 ^of protein, > 1.8 μg/cm^2 ^of hemoglobin and > 4 Log_10 _cfu/cm^2 ^bioburden remaining.

The volume of fluid to be flushed through channels that is required by endoscope manufacturers for the detergent and rinse stages of manual cleaning is large. Olympus recommends 90 mLs per suction-biopsy channel for colonoscopes and according to Puszko [12] 150 mLs per channel is recommended by the Australian endoscope reprocessing guidelines. For manual cleaning this requires staff to use syringes filled with fluid and repeated manual flushing that often leads to repetitive strain injuries [12]. The use of stand alone pumps to facilitate the flushing of cleaning and rinsing fluid has become widely accepted in centres that can afford these pumps [12]. The use of endoscope reprocessors with validated cleaning cycles would greatly reduce the strain on staff and would standardize the fluid volume being flushed.

Despite the overall low rates of infection associated with flexible endoscopy procedures, flexible endoscopes are still the most common cause of healthcare device associated outbreaks [29]. Bisset et al [4] have showed that the incidence of residual microbial contamination for patient ready gastroscopes and duodenoscopes was 1.9% and 1.8% respectively. Furthermore, they indicated that the incidence of bioburden contamination increased with the number of times the endoscope was used. This suggests that biofilm may form over repeated uses and this may be related to the wide variation in the manual cleaning performed [6,4]. The recent study by Alfa and Howie [30] modelling build-up biofilm as well as the one by Vickery et al [31] using endoscope tubing both confirm that repeated rounds of exposure to organic material, cleaning and HLD (complete reprocessing) result in an accumulation of material within the channel. The rate of accumulation is modulated by organic load [30] and type of detergent used [17]. The review by Moses and Lee [6] reveal that only 70% of centers surveyed flushed detergent through channels and used brushes for the channels and valves (30% omitted manual cleaning and relied on the AER to perform cleaning even though there were no AERs at that time with FDA clearance for cleaning cycles). Marion et al [32] suggest that periodic use of biofilm detaching agent may be needed to reduce the risk of biofilm accumulation in scope channels. These studies all suggest that in healthcare there is widespread difficulty in achieving the endoscope manufacturers recommended manual cleaning and that great variability exists in the manual cleaning currently being performed.

The value of AERs with validated cleaning combined with efficient HLD and final rinsing cannot be overemphasized. However, not all AER cleaning cycles are equivalent as many of the "cleaning" cycles in commercially available AERs do not have FDA cleared cleaning claims and therefore still require manual cleaning prior to processing in the AER. As indicated by Alfa and Howie [30] when an aldehyde is used for HLD (as is the case for ECR) it becomes critical that the cleaning needs to be thorough and reproducible to reduce the risk of microbial survival in accumulated organic material. Our data suggest that because of the efficacy of the ECR cleaning cycle and the fact that the cycle cannot be "shortened" by the user, the potential for build-up biofilm would likely be reduced compared to manual cleaning where short-cuts in cleaning are common.

The cleaning efficiencies achieved by the AdaptaScope and LS 2000 AERs tested by Kircheis and Martiny [33] using endoscope channel tubing indicated that efficient microbial removal was only achieved when direct channel connection was used. The two "pressure chamber" models of AERs evaluated could not achieve flow through of the inoculated tubing therefore there was no bioburden reduction. The ECR evaluated in the current study uses direct channel connection and the data from our simulated-use study demonstrated that the RF achieved by the cleaning cycle alone was > 6 for all surface sites and > 4.5 for all air/water, suction/biopsy and auxillary water channels for all types of flexible endoscopes. This further extends Kircheis and Martiny's [33] work on tubing segments to demonstrate how efficient cleaning can be achieved in the various channels and surface of flexible endoscopes. The ECR was also able to achieve an enterococcus RF of > 4 for the elevator guidewire channel which presents unique challenges to cleaning which were not addressed in Kircheis and Martiny's [33] study. Indeed this RF of 4.01 achieved by the ECR cleaning cycle is superior to a previous report [22] for the Reliance AER (RF of 2.10) cleaning as well as for complete manual cleaning (RF of 3.22). The cleaning efficacy achieved using an AER with "boot pressure chamber" technology versus "direct channel connection" technology will depend entirely on the manufacturer's specifications for that particular machine. Both technologies have advantages and disadvantages. Direct channel connection allows for monitoring of individual channel flow (although not all AERs with channel connections provide this option) whereas this is not currently available for "boot pressure chambers". Boot pressure chamber overcomes possible user errors with channel connection mix-ups that may occur with traditional AERs that use direct channel connections. Users need to determine which AER process best suites their needs and should thoroughly assess the validation data provided for any AER they are considering.

It should be noted that it is difficult to assess the effects of lumen anomalies such as scratches or biofilm buildup on the efficacy of either manual or ECR cleaning. However, the in-use study using patient-used endoscopes does provide valuable data on cleaning efficacy for devices known to have been exposed to normal wear and tear within the clinical setting.

Although only Olympus flexible endoscopes were included in the current study, the data from this study provides "proof of concept" that the cleaning technology of the ECR is adequate. In the study by Alfa et al [22] where Olympus, Pentax and Fujinon endoscopes were evaluated [22] the AER cleaning cycle showed similar cleaning efficiencies regardless of which manufacturer's endoscopes were tested. In addition, as the ECR manufacturer completes validation of different scope types and models they will be added to their list of validated endoscopes that can be processed through the ECR.

## Conclusions

In summary the data presented for the simulated-use testing and clinical use testing clearly demonstrate that the cleaning cycle does provide reliable organic and bioburden removal from 98.8% of surfaces and 99.7% of lumens for the bronchoscopes, duodenoscopes, gastroscopes and colonoscopes that were tested. The ECR cleaning for endoscope surfaces and channels is superior to optimal manual cleaning providing all the ECR manufacturer's specific instructions are followed (including the type of enzymatic detergent used in the ECR). Although only Olympus flexible endoscopes were evaluated in this study it does provide stringent proof of concept for the cleaning phase of the ECR complete reprocessing cycle (cleaning and HLD). This cleaning efficiency for patient-used endoscopes is only validated for elective procedures (not emergency endoscopies) and only if the bedside pre-cleaning is done and the transit time is less than an hour prior to placing the endoscope into the ECR.

## Competing interests

The ECR and all funds for this study were provided by Advanced Sterilization Products, a Division of Ethicon, a Johnson and Johnson company. MJA has undertaken contract research projects (unrelated to the current publication) for a variety of companies including; 3M, bioMerieux, Olympus, Case Medical, Intelligent Hospital Systems, Johnson & Jonson, Novaflux, STERIS and Virox. No monies from the current or past research contracts have gone to MJA, they were administered through the St. Boniface Research Centre and were used for research related expenses only. MJA has been a sponsored conference speaker and she has acted as a paid consultant for preparation of a one time literature review for STERIS in 2003 and for providing a one time educational microbiology workshop for 3M staff in 2006.

PD, NO and IF have not acted as consultants and have no financial or other link with any company.

## Authors' contributions

All authors have read and approved the final manuscript. MJA was responsible for the conception of the research project and provided direction for the experimental testing. Writing of the protocols, performing sample collection, all testing and data entry were performed by PD, NO and IF. MJA was responsible for data analysis and manuscript preparation with input from PD, NO and IF.

No company played any role in the gathering or preparation of data. Advanced Sterilization Products provided input on the study design and editing of the manuscript.

## Pre-publication history

The pre-publication history for this paper can be accessed here:

http://www.biomedcentral.com/1471-2334/10/200/prepub
