# Correction: EVOTECH^® ^endoscope cleaner and reprocessor (ECR) simulated-use and clinical-use evaluation of cleaning efficacy

**DOI:** 10.1186/1471-2334-10-291

**Published:** 2010-10-04

**Authors:** Michelle J Alfa, Pat DeGagne, Nancy Olson, Iram Fatima

**Affiliations:** 1 Diagnostic Services of Manitoba, 409 Tache Avenue, Winnipeg, MB, R2 H 2A6, Canada; 2 St. Boniface Research Centre, 351 Tache Avenue, Winnipeg, MB, R2 H 2A6, Canada; 3 University of Manitoba, Winnipeg, MB, 745 Bannatyne Avenue, Room 543 Basic Medical Sciences Building, R3E 0J9, Canada

## Correction

The original submission of this manuscript included a citation of one of the authors' patents [1]. During the review process, we were alerted to the existence of another relevant patent. As is the norm, it was our intention that all references to any patents should be removed from the manuscript and replaced by the relevant original peer reviewed research. Unfortunately, the authors were not asked to do this and the manuscript was inadvertently published with the patent citation included.

In the interests of fairness, we cite the other patent in question in this Correction [2].

We fully acknowledge that this was an editorial error and apologize to the authors and reviewers for any inconvenience this has caused them.

## Pre-publication history

The pre-publication history for this paper can be accessed here:

http://www.biomedcentral.com/1471-2334/10/291/prepub
